# Uptake of antiretroviral therapy among people living with HIV in Addis Ababa, Ethiopia: A qualitative exploratory study

**DOI:** 10.4102/hsag.v31i0.3457

**Published:** 2026-09-21

**Authors:** Ayehu M. Belay, Hulisani Matakanye, Mokgadi C. Matlakala

**Affiliations:** 1 Department of Health Studies, College of Human Sciences, University of South Africa, Pretoria, South Africa

**Keywords:** antiretroviral therapy, differentiated service delivery, HIV, barriers, facilitators, adherence, Ethiopia, qualitative study

## Abstract

**Background:**

Unsuppressed viral load despite antiretroviral therapy (ART) remains a major challenge to effective HIV treatment in Ethiopia. In Addis Ababa, retention in care and adherence remain problematic despite the widespread implementation of differentiated service delivery (DSD) models.

**Aim:**

To explore ART uptake among people living with HIV (PLHIV) in Addis Ababa from the perspectives of PLHIV and healthcare providers, using a qualitative exploratory approach.

**Setting:**

The study was conducted in eight selected public health centres in Addis Ababa, Ethiopia.

**Methods:**

A qualitative exploratory study design was employed, involving in-depth interviews with a purposive sample of 36 participants: 20 people living with HIV and 12 healthcare providers (including ART nurses, pharmacists and HIV programme coordinators). Data were analysed thematically to investigate key barriers and facilitators influencing ART uptake.

**Results:**

Six themes emerged: (1) structural and systemic barriers, including drug shortages and transportation challenges; (2) psychosocial-related barriers, such as stigma and fear of disclosure; (3) variable awareness and perceptions of DSD models; (4) adherence motivators, including health preservation and social support; (5) provider–patient relationship dynamics; and (6) recommendations focusing on health-system reforms and community engagement. Limited awareness of DSD options and inconsistent implementation reduced the effectiveness of DSD models.

**Conclusion:**

Improving ART delivery in Addis Ababa requires integrated interventions that address structural, economic and psychosocial barriers, strengthen patient–provider communication and enhance community engagement.

**Contribution:**

The study provides actionable, context-specific evidence on barriers and facilitators to ART uptake, supporting efforts to improve adherence and retention and to advance progress towards global HIV targets and Sustainable Development Goals 3.3 and 3.8.

## Introduction

HIV remains a major global public health challenge. In 2024, approximately 40.8 million people were living with HIV worldwide, with about 630 000 AIDS-related deaths reported (Joint United Nations Programme on HIV/AIDS [UNAIDS] 2024, 2025). Although substantial progress has been made in expanding access to antiretroviral therapy (ART), significant inequities in uptake and retention persist, particularly in sub-Saharan Africa, which continues to bear the greatest burden of the epidemic. Ethiopia faces similar challenges, particularly in urban settings. According to the Ethiopian Public Health Institute (EPHI 2024), approximately 603 537 people were living with HIV in 2023, with Addis Ababa accounting for over 110 000 cases and an estimated prevalence of 3.42%. Although ART services are provided free of charge, suboptimal uptake and inconsistent engagement in care remain significant concerns. These gaps contribute to treatment failure, preventable morbidity and continued HIV transmission. Evidence indicates that barriers to ART uptake and retention include stigma, fear of disclosure, inadequate partner and social support, limited awareness of treatment benefits and structural challenges such as transportation costs, long waiting times and competing livelihood priorities (Gizaw, Boke & Geremew 2021; Kassaw et al. 2022). Multiple stakeholders influence ART uptake and retention in Addis Ababa. People living with HIV (PLHIV) and their families make critical decisions regarding testing, disclosure, adherence and service utilisation, often amid stigma and economic pressure, which remain key barriers to sustained engagement in care (Ameyaw et al. 2024). Healthcare providers in public facilities deliver ART, counselling and follow-up care under conditions shaped by structural and health system constraints, including workload and service accessibility challenges that affect continuity of care (Mbuagbaw et al. 2024). Sub-city HIV/AIDS programme coordinators and health system managers play a critical role in organising services and implementing differentiated service delivery (DSD) models designed to improve retention and client-centred care (Merid et al. 2024). Community-based organisations, peer groups and civil society actors provide essential social support, stigma-reduction interventions and linkage to care, which have been shown to significantly enhance ART adherence and re-engagement in care, particularly among vulnerable populations (Ameyaw et al. 2024; World Health Organization Eastern Mediterranean Regional Office [WHO EMRO] 2024). In response to persistent barriers, Ethiopia introduced DSD models in 2017 as part of its national ART programme (Ministry of Health, Ethiopia 2017). Differentiated service delivery is a client-centred approach that tailors HIV services to the diverse needs of PLHIV to improve access, retention and quality of care while enhancing health system efficiency (Grimsrud & Wilkinson 2021). Key strategies include multi-month dispensing of ART, decentralised and community-based drug distribution and peer-led adherence support mechanisms. These approaches aim to reduce structural and psychosocial barriers to care. Evidence from sub-Saharan Africa suggests that DSD models can enhance ART uptake and continuity by reducing travel burden, minimising opportunity costs, decreasing stigma associated with frequent facility visits and strengthening patient–provider relationships (Long et al. 2020). At the health system level, DSD contributes to reduced facility congestion, improved allocation of human resources, and better management of patients with complex clinical needs (Ntjikelane et al. 2025). However, the availability of DSD alone does not ensure optimal ART uptake. Persistent challenges – including medication stockouts, limited patient awareness of service options, inconsistent implementation across facilities and ongoing stigma – continue to limit programme effectiveness (Merid et al. 2024). Despite these realities, there remains limited context-specific qualitative evidence that explores how PLHIV and healthcare providers perceive and experience ART uptake within Addis Ababa. In particular, the lived experiences of participants, including challenges in initiating ART, maintaining adherence, navigating DSD models and interacting with healthcare providers – are not sufficiently documented. This gap limits the ability to design responsive, patient-centred interventions that address both behavioural and system-level barriers. Given the critical role of ART in improving survival, enhancing quality of life and preventing HIV transmission, improving ART uptake is essential for Ethiopia’s progress towards achieving the UNAIDS 95-95-95 targets and Sustainable Development Goal 3 (targets 3.3 and 3.8) (Seid Ebrahim et al. 2025; UNAIDS 2020). Therefore, this study aims to explore the factors influencing ART uptake among PLHIV in Addis Ababa, Ethiopia, from the perspectives of both PLHIV and healthcare providers, using a qualitative exploratory approach, with particular attention to participant-level experiences, stakeholder roles and health system dynamics.

## Research methods and design

### Study design

This study employed a qualitative exploratory design to investigate barriers to the uptake of differentiated ART service delivery (DSD) models among PLHIV. It also explored healthcare providers’ perceptions of DSD implementation and delivery in the urban context of Addis Ababa.

### Study setting

The study was conducted in Addis Ababa, the capital of Ethiopia, which has an estimated population of approximately 5.2 million and is projected to reach 7.5 million by 2030 (World Bank 2021). The city is ethnically and linguistically diverse, comprising more than 80 groups, including Amhara, Oromo, Gurage and Tigray populations (Central Statistical Agency, Ethiopia 2020). Addis Ababa bears a substantial HIV burden, with an estimated prevalence of 3.4%, influenced by factors such as urbanisation, migration, socioeconomic inequality, stigma and health-seeking behaviours, including reliance on traditional healing practices (EPHI 2022). Eight public health centres providing ART services were purposively selected from four sub-cities, Addis Ketema, Kirkos, Bole and Gulele, to ensure representation of diverse urban contexts. The selected sites were Addis Ketema Health Centre (AKLHC), Felega Melese Health Centre (FMHC), Bole 17 Health Centre (B17HC), Kirkos Health Centre (KKHC), Gotera Health Centre (GTHC), Saris Health Centre/Selam Health center (SMHC) and Hidase Health Centre (HGHC). The selection was based on predefined criteria, including variation in patient load (high- and low-volume facilities), geographic distribution and differences in service, delivery experiences, including the implementation of DSD models. This purposive sampling approach was intended to capture a wide range of perspectives and contextual factors influencing ART uptake across heterogeneous urban settings, rather than for convenience.

### Study population

The study population consisted of two groups: all adults aged 18 years and above living with HIV (PLHIV), who had been enrolled in ART for at least 12 months, and all healthcare providers involved in HIV care and ART service delivery, including physicians, nurses and pharmacists in Addis Ababa. Including both PLHIV and healthcare providers enabled the triangulation of perspectives across multiple levels of influence on ART uptake and continuity of care. Specifically, PLHIV provided insights into individual-level factors, such as personal beliefs, experiences and adherence behaviours; healthcare providers contributed perspectives on health system-level factors, including service delivery practices, resource availability and implementation of DSD models, while both groups offered reflections on community-level factors, such as stigma, social support and cultural norms influencing engagement in care.

### Sample size and sampling procedure

A purposive sampling approach, combining maximum variation and criterion-based strategies, was used to recruit participants with relevant experience in ART service delivery and utilisation in Addis Ababa. Initially, a total of 40 participants were targeted at four sub-cities, Addis Ketema, Bole, Kirkos and Gulele, including 24 PLHIV (3 per facility), 12 healthcare providers and 4 sub-city HIV/AIDS programme coordinators. Two health centres were selected within each sub-city to capture variation in ART client load (high- and low-volume facilities). A stratified purposive sampling approach was used to recruit PLHIV from each facility, ensuring diversity in age, gender and treatment experience. Healthcare providers were purposively selected to represent different professional roles, including nurses, pharmacists and physicians, while programme coordinators were included to provide system-level perspectives. A convenience element was applied, whereby eligible participants attending ART clinics during the data collection period were invited to participate. The final sample comprised 36 participants, including 20 PLHIV, 12 healthcare providers and four programme coordinators, after adjustments due to participant availability. Recruitment across sites varied slightly, with PLHIV ranging from 1 to 3 participants per facility, while one coordinator and three healthcare providers were included per sub-city.

The sample size was guided by the principle of information power, and data collection continued until thematic saturation was achieved (Saunders et al. 2018, 2023).

### Purpose and objectives of the study

The purpose of this study was to explore barriers to the uptake of differentiated ART service delivery (DSD) models among people living with HIV (PLHIV) and to examine healthcare providers’ perceptions of the implementation of DSD models in Addis Ababa.

This study was guided by the following objectives:

- To explore the barriers affecting the uptake of differentiated ART service delivery (DSD) models among PLHIV in Addis Ababa.
- To explore healthcare providers’ perceptions of the implementation and delivery of differentiated ART care models in Addis Ababa.

### Data collection procedure

Pilot interviews were conducted to refine the semi-structured interview guide (Annexure A). Ethical clearance and administrative permission were obtained from the relevant institutional review board and the participating health facilities. Participants were identified through clinic registers and in collaboration with nurses and ART coordinators and were invited directly by the principal researcher for interview scheduling. No posters or mass announcements were used. Data were collected through individual, in-depth, semi-structured interviews with 20 PLHIV and 12 healthcare providers between October and November 2024. Interviews lasted approximately 40 min – 60 min and were conducted in private rooms within the health facilities to ensure privacy and minimise disruption to clinic duties. People living with HIV were interviewed before healthcare providers to reduce potential influence on staff responses. Written informed consent was obtained from all participants, and the researcher explained the study purpose, procedures, risks and benefits and participants’ rights, including the right to withdraw at any time. Interviews were audio-recorded with consent, and supplementary field notes were taken during and immediately after each interview. Each participant was assigned a unique identifier to maintain anonymity. Interviews were transcribed verbatim in Amharic and translated into English, with bilingual verification to ensure accuracy (Squires 2009). Thematic analysis was conducted using ATLAS.ti version 24, involving repeated reading, coding and development of themes and sub-themes. Reflective memos were maintained to enhance reflexivity and methodological rigour (Lincoln & Guba 1985). Verbatim quotations were used to illustrate key themes.

### Data management and analysis

All qualitative data were managed in accordance with the data protection and research ethics protocols of the relevant institution (information redacted to maintain the integrity of the review process). Audio-recorded interviews were transcribed verbatim, checked for accuracy against the recordings and imported into ATLAS.ti version 24. Audio files, transcripts, field notes and reflective memos were stored on a password-protected computer, accessible only to the principal researcher and supervisors; any identifiable information was removed to ensure confidentiality and anonymity. Data were analysed using reflexive thematic analysis following Braun and Clarke (2006). ATLAS.ti version 25 (ATLAS.ti Scientific Software Development GmbH, Berlin, Germany) was used to organise transcripts, manage an evolving codebook, record analytic memos and retrieve coded text. An independent co-coder coded a subset of transcripts; discrepancies were resolved through consensus meetings informed by data excerpts and analytic memos. Data were analysed using reflexive thematic analysis following Braun and Clarke (2006). ATLAS.ti (version 25) served as computer-assisted qualitative data analysis software (CAQDAS) to organise transcripts, manage an evolving codebook, record analytic memos and retrieve coded text. A trained independent co-coder independently coded a purposive subset of *n* = *X* transcripts. Code application and theme development were iteratively compared within ATLAS.ti and discussed in consensus meetings; disagreements were resolved by referring to raw transcripts and memos, leading to agreed revisions of code definitions and final themes. Final themes were illustrated with verbatim quotations to preserve participants’ perspectives.

### Ethical considerations

Ethical clearance to conduct this study was obtained from the University of Pretoria College of Human Sciences Research Ethics Review Committee (No. Rec-240816-052) and Addis Ababa Health Bureau (No. A/A/H/14521/227). Administrative permission was also granted by the respective facility managers. All ethical principles, including respect for people, beneficence, non-maleficence and justice, were strictly observed throughout the research process. Participants received comprehensive information about the study purpose, procedures, potential risks and benefits and their rights. Written informed consent was obtained from all participants prior to data collection. Participation was voluntary, and participants were informed of their right to decline or withdraw at any time without affecting their access to HIV services or clinical care. Confidentiality and anonymity were ensured by not collecting personal identifiers; participants were assigned unique codes. Audio recordings and transcripts were stored on a password-protected computer accessible only to the principal researcher and academic supervisors. Interviews were conducted in private rooms, and verbatim quotations were anonymised in reporting. The study aimed to contribute to improved HIV care and ART services in Addis Ababa, offering potential indirect benefits to people living with HIV (PLHIV) and the health system. The main risk was emotional discomfort when discussing sensitive topics such as stigma, disclosure and treatment challenges. Participants could skip questions, pause or withdraw at any time, and contact details for psychosocial support services were provided where appropriate. The study complied with the *Protection of Personal Information Act (POPIA)* and relevant data-protection standards through lawful processing, purpose limitation, data minimisation and secure storage. Trustworthiness was enhanced through semi-structured in-depth interviews, immediate verbatim transcription with bilingual verification, peer debriefing, co-coding by an experienced qualitative researcher, reflexive memos and the use of verbatim quotations. Researcher bias was addressed through reflexive memos, bracketing and regular discussion with supervisors; social-desirability bias was minimised by emphasising confidentiality and using nonjudgemental probing; selection bias was acknowledged, and findings were interpreted accordingly. These measures protected participants, upheld data integrity and strengthened the credibility of the study findings.

## Results

### Demographic characteristics of the participants

The study included 36 participants classified into two groups: people living with HIV (PLHIV) and healthcare providers (nurses, pharmacists and doctors). Most participants were females (66.7%). The detailed demographic characteristics of the participants are summarised in Table 1.

**TABLE 1:** Demographic characteristics of the study participants (*N* = 36).

Participant group	*n*	Sub-cities (*n*)	Age category (Years)	Male (*n*)	Female (*n*)	Primary role
Sub-City HIV/AIDS Programme Coordinators	4	Addis Ketema (1), Bole (1), Kirkos (1), Gulele (1)	25–34	1	1	HIV/AIDS Programme Coordinators
			35–44	1	1	
Healthcare Providers	12	Addis Ketema (3), Bole (3), Kirkos (3), Gulele (3)	25–34	2	3	Nurses, Pharmacists, Doctors
			35–44	1	4	
			45–55	1	1	
People Living with HIV (PLHIV)	20	Addis Ketema (5), Bole (4), Kirkos (5), Gulele (6)	25–34	1	4	PLHIV receiving ART care
			35–44	3	7	

45–55	2	3
**Total**	**37**	**Addis Ketema (9), Bole (8), Kirkos (10), Gulele (10)**	-	**12**	**24**	-

ART, antiretroviral therapy.

### Overview of findings

Analysis of the in-depth interviews resulted in six interrelated themes, reflecting individual, community- and family-level and health-system-level factors shaping ART uptake and continuity of care. These themes capture the complex interplay of barriers and facilitators within routine HIV service delivery, including the implementation and experience of DSD models in Addis Ababa.

### Thematic findings

Six major themes and corresponding sub-themes emerged from the analysis (see Table 2).

**TABLE 2:** Summary of themes and sub-themes.

Themes	Sub-themes
1. Structural and health system constraints limiting ART uptake and continuity of care	1.1. Medication stockouts and supply-chain disruptions 1.2. Financial and logistical challenges limiting access to care
2. Psychosocial burden and stigma as barriers to care engagement	2.1. Fear of disclosure and anticipated stigma 2.2. Emotional and mental health challenges affecting adherence
3. Perceived role of differentiated service delivery (DSD) models in improving ART access and utilisation	3.1. Convenience, reduced travel and enhanced privacy under DSD 3.2. Limited awareness and policy gaps in DSD implementation
4. Motivators that sustain ART adherence and engagement	4.1. Beliefs in health improvement, survival and treatment efficacy 4.2. Social and familial support as sources of strength
5. Patient–provider relationships shaping ART uptake experiences	5.1. Trust, partnership and positive communication in care 5.2. Gaps in communication and responsiveness within service delivery
6. Participant-informed strategies for improving ART uptake and service delivery	6.1. Health system level strengthening to improve service delivery 6.2. Community ‘driven’ interventions to reduce stigma and support PLHIV 6.3. Programmatic and institutional innovations to enhance DSD and adherence

ART, antiretroviral therapy.

### Interpretation of findings

The findings are presented thematically, drawing on the accounts and experiences of PLHIV and healthcare providers to explore how multiple interacting individuals, community and health-system-level factors shape ART uptake and continuity of care in Addis Ababa. Interpretation is embedded within the presentation of results to capture the complexity of lived experiences and to situate the findings within existing evidence on ART uptake and DSD. In this way, the article focuses explicitly on understanding the barriers and facilitators of ART uptake from the perspectives of PLHIV and healthcare providers, and how these factors influence routine HIV service delivery and the implementation of DSD models in an urban Ethiopian setting.

The six overarching themes that emerged are as follows:

#### Theme 1: Structural and health system constraints limiting antiretroviral therapy uptake and continuity of care

Structural and systemic barriers were the most pervasive impediments to consistent ART uptake across participant groups. Weaknesses in supply chain management, service organisation and resource allocation undermined timely access to medication and continuity of care, limiting the effectiveness of client-centred approaches such as DSD.

**Sub-theme 1.1: Medication stockouts and supply-chain disruptions:** Medication stockouts, particularly of prophylactic drugs such as cotrimoxazole, were repeatedly cited as a major barrier to sustained ART use. Interruptions in medication availability disrupted treatment routines, heightened anxiety and eroded trust in the health system. In response, some PLHIV reported rationing or reducing doses to extend limited supplies, practices that increase the risk of treatment failure and drug resistance.

One participant described her concern about potential medication shortages, explaining that she feared there might not be enough ART for all patients in the future.

She explained:

‘I can get more when my medicine runs out. I worry that if I take too much now, I may not have enough later, so I try to save some just in case’. (Female, PLHIV, P03)

This response illustrates how anticipated ART stockouts can lead to cautious or suboptimal self-management of medication, even among patients who are otherwise engaged in routine clinic care.

Healthcare providers acknowledged inter-facility redistribution as a temporary coping mechanism but emphasised its unsustainability, noting that it merely shifts shortages rather than addressing underlying supply chain weakness. These findings highlight how systemic inefficiencies directly undermine adherence behaviours and compromise the perceived reliability of ART services.

Another participant stated that:

‘When one clinic runs out of medicine, we try to help by sharing medicine from other clinics that have something extra. This helps patients for a while, but it is not a good long-term solution. It’s like trying to fix a leak by moving water elsewhere; the problem does not really go away and soon there will be shortages everywhere. To truly help patients, we need a better plan that prevents shortages from occurring in the first place’. (Female, Nurse, 30 years old)

**Sub-theme 1.2: Financial and logistical challenges limiting access to care:** Economic hardship and logistical constraints significantly limited clinic attendance and adherence.

Participants described long travel distances, high transport costs, prolonged waiting time and lost income as persistent barriers. For many, attending clinic appointments requires difficult trade-offs between healthcare and basic household needs.

One participant indicated that:

‘Sometimes I have to choose between paying for transport to the clinic or buying food for my children’. (Female, PLHIV, PL09)

Similarly, a participant explained:

‘The cost of transportation is very high for me. Sometimes, I have to decide whether to spend money on food for my children or to pay for the trip to the clinic. It’s a really tough choice, and it makes life very difficult’. (Female, PLHIV, PL13)

The interaction between financial strain and service inefficiencies contributed to missed appointments and treatment interruptions, reinforcing evidence that structural vulnerability undermines sustained ART engagement.

#### Theme 2: Psychosocial burden and stigma as barriers to care engagement

The study found that beyond material constraints, psychological factors played a central role in shaping ART uptake. Stigma, fear of disclosure and unmet mental health need discouraged consistent engagement in care and complicated adherence decisions.

**Sub-theme 2.1: Fear of disclosure and anticipated stigma:** Participants widely reported avoiding clinics near their homes to prevent inadvertent disclosure of their HIV status. Fears of gossip, judgement and social exclusion prompted some to travel long distances or skip appointments altogether.

One participant indicated that:

‘If people see me at the clinic, they start talking. Sometimes I miss appointments just to avoid being seen’. (Female, PLHIV, PL07)

Healthcare providers confirmed that confidentiality concerns frequently influenced patients’ care-seeking behaviours. These findings illustrate how stigma transforms routine care into a source of anxiety, reinforcing secrecy and undermining consistent ART uptake.

**Sub-theme 2.2: Emotional and mental health challenges affecting adherence:** Living with HIV was described as an ongoing emotional burden marked by anxiety, loneliness, depression and mental fatigue. These psychological challenges diminished motivation and reduced the capacity to maintain long-term adherence.

Another participant indicated that:

‘It’s not just about taking pills. It’s a daily struggle for the rest of your life’. (Female, PLHIV, PL17)

Providers emphasised that neglecting mental health needs weakens adherence efforts, underscoring the necessity of integrating psychosocial support into HIV care.

#### Theme 3: Perceived role of differentiated service delivery models in improving antiretroviral therapy access and utilisation

Participants generally viewed DSD models as important facilitators of ART uptake by reducing logistical burdens, protecting privacy and enhancing autonomy. However, uneven implementation and limited awareness constrained their full potential.

**Sub-theme 3.1: Convenience, reduced travel and enhanced privacy under differentiated service delivery:** Multi-month dispensing, fast-track refills and community-based ART delivery reduced clinic visit frequency, transportation costs and exposure to stigma.

One participant noted:

‘Now that I receive three months’ worth of medicine at one time, things have become much easier for me. I don’t have to keep going back to the clinic so often, which saves me a lot of time and money. It also helps me avoid unwanted attention – people used to ask why I was always at the clinic, and that made me uncomfortable. Now, I feel more at ease and can focus on living my life’. (Male, PLHIV, PL6)

Another participant added:

‘Now, I get my medicine for a longer period, so I don’t have to come back often. When I go to pick it up, I don’t have to wait in long lines. It makes the whole process more private and less stressful for me’. (Female, PLHIV, PL3)

From the providers’ perspective, DSD models also contributed to more manageable clinic workloads. Providers confirmed that DSD allowed them to dedicate more attention to patients with advanced illness and improve the overall quality of care.

Healthcare provider stated:

‘This makes our work easier and faster in the Differentiated Service Delivery (DSD) program. It helps reduce the number of patients we need to see often, so we have more time to focus on those who need extra care’. (Male, Doctor, 32 years old)

Another provider noted:

‘It’s good for patients because they don’t have to spend as much money or time traveling to the clinic for frequent visits. This makes it easier for them to keep up with their treatment’. (Male, Doctor, 32 years old)

**Sub-theme 3.2: Limited awareness and policy gaps in differentiated service delivery implementation:** Despite availability, many PLHIV were unaware of DSD eligibility or enrolment procedures. Providers acknowledged inconsistent communication and a lack of standardised implementation across facilities.

A participant articulated this gap by stating:

‘I’ve heard that some people receive medicine for three or six months at a time, but no one has told me if I can get it that way too. So, I just keep coming to the clinic every month like I always have’. (Female, PLHIV, PL9)

Similarly, other participants observed:

‘In our community, most people don’t know about these new ways to get HIV treatment. Many haven’t heard that there are easier or better options available’. (Female, PLHIV, PL12)

Participant further expressed uncertainty:

‘I’m not sure if I’m allowed to get the medicine for a longer time. No one has ever explained it to me, so I just keep coming to the clinic every month like before’. (Female, PLHIV, PL10)

Healthcare providers acknowledged these communication lapses. One provider noted:

‘We are not consistently educating patients about Differentiated Service Delivery options. Some staff do not bring it up unless the patient asks’. (Male, Senior Nurse, 38 years old)

Another provider emphasised the inconsistency in information dissemination:

‘We need to make sure Differentiated Service Delivery is introduced in the same way for everyone. Right now, it depends on which provider you meet and how busy they are. That means some people get more information or better options than others, which isn’t fair’. (Female, Nurse, 40 years old)

A program coordinator highlighted the oversight in community engagement:

‘We spent a lot of effort setting up the DSD system, but we didn’t realise how important it was to educate the community about it. Many clients still don’t know about the different options available to them’. (Male, Program Coordinator, 33 years old)

#### Theme 4: Motivators that sustain antiretroviral therapy adherence and engagement

The current study discovered that despite substantial barriers, participants identified powerful motivators that sustained adherence, particularly beliefs in treatment effectiveness and strong social support.

**Sub-theme 4.1: Beliefs in health improvement, survival and treatment efficacy:** This study further found that belief in ART as life-sustaining and observable health improvements reinforced adherence and fostered hope.

One participant stated that:

‘ART is what keeps me alive’. (Female, PLHIV, P11)

This study noted that such convictions strengthened resilience and commitment to lifelong treatment.

**Sub-theme 4.2: Social and familial support as sources of strength:** Emotional, instrumental and informational support from family, peers and healthcare providers played a critical role in sustaining adherence. Participants emphasised encouragement, reminders, material assistance and shared experiences as sources of motivation.

Participant indicated that:

‘[*M*]y family and support group give me strength when I feel like giving up’. (Female, PLHIV, PL12)

#### Theme 5: Patient–provider relationships shaping antiretroviral therapy uptake experiences

This study further found that the quality of provider-patient relationships emerged as a decisive factor influencing adherence, retention and satisfaction with care.

**Sub-theme 5.1: Trust, partnership and positive communication in care:** The findings of the study show that respectful communication, empathy, confidentiality and shared decision-making fostered trust and encouraged sustained engagement.

One participant explained:

‘Having a health provider who understands me makes all the difference. She listens to my concerns, and I feel like she’s genuinely interested in my health. That makes me trust her more and stick to my treatment’. (Female, PLHIV, PL5)

Other Participant added:

‘When my doctor remembers my name and knows my story, it makes me feel respected and cared for. That personal connection gives me more confidence and motivation to keep up with my treatment because I know I’m not just another patient, I’m someone who matters’. (Female, PLHIV, PL13)

Similarly, Participant noted:

‘My doctor really listens to me and includes me in making decisions about my care. It feels like we’re working together as partners, not like I’m being told what to do without a say. That makes me feel respected and more in control of my treatment’. (Female, PLHIV, PL15)

**Sub-theme 5.2: Areas requiring improvement:** Study found that overcrowding, limited consultation time, frequent staff rotation and inconsistent management of side effects undermined continuity of care and weakened trust.

A participant stated that:

‘Clinics are always full of people, and there is no time to talk about how the medicine affects me. I leave feeling like what I say doesn’t really matter’. (Female, PLHIV, PL3)

Similarly, other participants remarked:

‘Sometimes visits feel rushed and I don’t get to talk about my side effects. It makes me hesitant to return’. (Male, PLHIV, PL6)

Another participant further explained:

‘I’ve seen five different providers this year. None of them knows my story; it’s like starting from the beginning every time’. (Male, PLHIV, PL18)

Frequent changes in healthcare providers disrupt the continuity of care and weaken the patient–provider relationship, thereby reducing patients’ sense of support and adherence to treatment.

Healthcare providers also acknowledged the challenges posed by high patient volumes.

Provider noted:

‘We manage more than 100 patients daily. There is just not enough time to give everyone the attention they need’. (Female, Nurse, 30 years old)

Provider emphasised the need for provider training:

‘Providers need refresher training to slow down and empathise. Even small gestures, eye contact, a pause, can rebuild trust’. (Male, Doctor, 34 years old)

According to a provider:

‘We need updated protocols for side effect management. Right now, it’s ad hoc and patients suffer silently’. (Male, Pharmacist, 34 years old)

Both patients and providers attributed these challenges to systemic constraints rather than individual failings.

#### Theme 6: Participant-informed strategies for improving antiretroviral therapy uptake and service delivery

Participants across all groups articulated clear, experience-based recommendations aimed at strengthening ART uptake and continuity of care in Addis Ababa. These recommendations reflect an understanding of ART challenges at multiple levels and emphasise the need for integrated, patient-centred and system-responsive interventions. Three interrelated sub-themes emerged: strengthening health system capacity, enhancing psychosocial and community-based support and optimising DSD implementation.

**Sub-theme 6.1: Health system-level strengthening to improve service delivery:** The study revealed that overcrowding, limited consultation time, frequent staff rotation and inconsistent side effect management undermined continuity of care and eroded trust.

A participant stated:

‘Even when patients are motivated, stockouts discourage them. We need a system that guarantees medicines are always available’. (Male, Pharmacist, 34 years old)

People living with HIV participants also emphasised the importance of improving clinic efficiency, including shorter waiting times and better appointment scheduling, to reduce opportunity costs associated with clinic visits:

‘If services were faster, more people would come regularly and not miss their appointments’. (Female, PLHIV, PL10)

These recommendations underscore the central role of health-system reliability in sustaining long-term ART engagement and highlight the need for structural reforms to support patient-centred care.

**Sub-theme 6.2: Community-‘driven’ interventions to reduce stigma and support people living with HIV:** Participants consistently emphasised the importance of psychosocial and community-based interventions to address stigma, emotional distress and social isolation. People living with HIV called for expanded counselling services, peer support groups and community education initiatives to normalise HIV care and reduce fear of disclosure.

Peer-led support mechanisms were described as particularly valuable for providing emotional encouragement, practical advice and shared experiences that strengthen adherence motivation.

One participant indicated that:

‘When you talk to someone who lives like you, you feel stronger and less alone’. (Female, PLHIV, PL14)

Healthcare providers recommended strengthening linkages between health facilities and community structures, including faith-based organisations, civil society groups and local leaders, to promote a supportive environment for ART uptake.

One care provider indicated that:

‘ART is not only a medical issue; it needs community acceptance and mental support’. (Female, Nurse, 33 years old)

Participants further emphasised the need to integrate mental health screening and counselling into routine HIV services to address depression, anxiety and treatment fatigue, which were seen as major but under-addressed barriers to sustained adherence.

**Sub-theme 6.3: Programmatic and institutional innovations to enhance differentiated service delivery and adherence:** Although DSD models were widely viewed as beneficial, participants highlighted gaps in awareness, eligibility assessment and consistency of implementation. Both PLHIV and healthcare providers recommended standardising DSD enrolment procedures and improving communication about available service options.

People living with HIV participants emphasised the need for clear, proactive information about DSD models, including eligibility criteria and benefits, to enable informed decision-making:

‘If patients are not told about these options, they cannot benefit from them’. (Male, PLHIV, PL06)

Healthcare providers recommended additional training and clear national guidance to support consistent DSD implementation across facilities:

‘Some facilities apply DSD very well, others do not. We need uniform guidance and follow-up’. (Male, Senior nurse, 38 years old)

Participants also suggested expanding digital health solutions, such as appointment reminders and electronic tracking systems, to support DSD and reduce missed visits. Transport subsidies and flexible refill schedules were proposed to further reduce economic barriers, particularly for vulnerable populations.

## Discussion

This study examined the influence of structural, socioeconomic and psychosocial factors on ART uptake and the experiences of participants on DSD in Addis Ababa. The findings reveal a dynamic interplay between health system conditions, individual circumstances and service-delivery arrangements, which is consistent with emerging research on ART programmes in Ethiopia and across sub-Saharan Africa. Medicine stockouts emerged as one of the most severe challenges reported by participants. Several PLHIV described treatment interruptions, rationing of medication and rising anxiety about continuity of care. For example, one female PLHIV (P03) explained that she tried to ‘save’ medicines in case of future shortages, illustrating how anticipated stockouts translate into suboptimal self-management of ART. These experiences align with national assessments indicating persistent supply-chain gaps in Ethiopia, despite the introduction of digital tracking systems such as the Dagu Elmis (Ministry of Health, Ethiopia 2023). A 2025 WHO assessment also reported that although medicine availability improved in some facilities, infrastructural limitations and staff shortages continue to compromise consistent pharmaceutical supply (WHO 2025). These system-level gaps are further reinforced by a 2022 Africa-wide DSD review, which identified antiretroviral (ARV) drug stockouts and supply-chain inconsistencies as major obstacles to programme implementation and patient outcomes (Rosen et al. 2022). Financial and logistical constraints, such as high transport costs, long travel distances and long waiting times, were repeatedly described by participants as barriers to sustained ART uptake. People living with HIV reported that frequent clinic visits disrupted their livelihoods and made it difficult to adhere consistently. A recent scoping review on ART adherence across sub-Saharan Africa similarly documented transport costs, income instability and poor clinic accessibility as major socioeconomic barriers impacting adherence (Merid et al. 2024). The structural vulnerabilities described by participants in this study therefore reflect broader systemic issues that continue to undermine HIV programme performance in resource-limited settings. Fears of disclosure and anticipated stigma were also prominent concerns. Several participants reported avoiding clinic visits close to their home communities or seeking care in distant facilities to avoid being recognised. One male PLHIV (PL06) noted that he preferred to collect his medicines from a facility further away ‘so that people in my neighborhood do not know I am on ARVs’. This pattern is consistent with evidence from Ethiopia showing a strong association between perceived stigma, non-disclosure and reduced engagement in HIV care (Kassaw et al. 2022). Stigma has similarly been cited as a major barrier to the scale-up of DSD models across sub-Saharan Africa, influencing care-seeking behaviour and discouraging uptake of community-based ART services (Armstrong-Mensah et al. 2022; UNAIDS 2023). Emotional and mental health burdens, including depression, loneliness and chronic stress, were described by many PLHIV as complicating their ability to adhere consistently. Several participants linked their mood and emotional state to treatment fatigue and missed doses. A scoping review on ART adherence across sub-Saharan Africa identified mental health challenges as one of the most critical individual-level barriers to sustained ART adherence in the region (Merid et al. 2024). The convergence of stigma and psychological distress in this study underscores the necessity of integrating mental health screening and counselling into routine HIV services, as participants explicitly called for such support. Participants expressed strong support for DSD models, noting that multi-month dispensing, fast-track refills, and community-based delivery reduced transportation barriers, saved time and minimised stigma exposure. Several PLHIV highlighted that collecting medicines every 3 or 6 months allowed them to maintain their daily routines and to avoid frequent clinic visits where they feared being identified as HIV-positive. National programme data support these perceptions: according to the Ministry of Health, Ethiopia (2023), in 2023, 53% of people on ART were enrolled in 6-month dispensing and 37% in 3–5-month dispensing, demonstrating significant progress in expanding convenient, patient-centred DSD options. This reinforces the growing consensus that DSD models reduce health-system burden while improving patient autonomy and retention. However, the study also revealed critical information and policy gaps. Many participants lacked clear information about DSD eligibility criteria and the range of available service options, and some were unsure whether they qualified for multi-month dispensing or community-based refills. Several PLHIV reported that clinic staff provided inconsistent or incomplete explanations. These findings are consistent with a 2022 Africa-wide review, which identified low health literacy among both patients and providers, along with inconsistent implementation of DSD models, as major barriers to maximising their benefits (Armstrong-Mensah et al. 2022). This indicates an urgent need for strengthened communication, standardised guidelines and structured provider training on DSD. These findings are consistent with a 2022 Africa-wide review, which identified low health literacy among both patients and providers, along with inconsistent implementation of DSD models, as major barriers to maximising their benefits (Armstrong-Mensah et al. 2022). This indicates an urgent need for strengthened communication, standardised guidelines and structured provider training on DSD. Despite structural and psychosocial barriers, some participants described powerful intrinsic motivators that supported their adherence. These included belief in ART effectiveness and visible health improvements, such as regained weight, increased energy and fewer opportunistic infections. For example, one male PLHIV (PL08) said that seeing his own health improve gave him ‘the strength to continue taking the pills every day’. This mirrors recurring evidence showing that perceived treatment efficacy and improved well-being significantly enhance adherence and foster hope for the future (Buh et al. 2023). Social and familial support also played a pivotal role in sustaining adherence. Participants reported that encouragement from family members, peers or support groups helped them stay motivated, remember to take their medicines and seek care during stressful periods. Peer groups in particular were described as sources of emotional reassurance, practical assistance and mutual accountability. Community-based support models have similarly been shown to strengthen adherence by fostering solidarity and reducing the isolation often associated with living with HIV (Thalia 2024). Participants emphasised the critical role of trust, empathy and respectful communication in supporting ART adherence. They valued consultations in which providers listened attentively, explained treatment clearly and treated them with dignity. Several PLHIV linked these relational qualities to their willingness to return to clinic and to disclose challenges with adherence. Supportive provider–patient relationships are well-documented facilitators of ART retention and patient engagement. Studies demonstrate that respectful, continuous communication strengthens patient motivation and improves treatment outcomes (Buh et al. 2023). The authors have carefully edited the manuscript for English language, grammar and academic style in accordance with Health SA Gesondheid (HSAG) author guidelines. However, the study also highlighted challenges such as brief consultations, clinic overcrowding and frequent staff rotations, which diminished continuity of care and made it difficult to build sustained therapeutic relationships. Many PLHIV reported feeling rushed during visits or consulting different providers each time, which undermined trust and reduced opportunities for in-depth discussions about adherence. Ethiopia’s 2024–2030 Human Resources for Health Strategy similarly identifies staffing shortages and uneven workforce distribution as systemic constraints affecting service quality (Ministry of Health, Ethiopia 2024). These weaknesses contribute to fragmented care and hinder the development of the sustained provider–patient relationships that are essential for long-term adherence and effective implementation of DSD models.

### Limitations of the study

This study was conducted among people living with HIV (PLHIV) and healthcare providers within selected urban healthcare facilities in Addis Ababa. As a result, the findings may not be transferable to all regions of Ethiopia or to rural settings, where health-system resources, cultural contexts and service-delivery arrangements may differ. The sample was drawn from urban public facilities with relatively better infrastructure, higher staffing capacity and more established DSD models than many rural facilities. Inclusion of sites from other regions or rural areas may have yielded different perspectives and a broader variation in experiences with ART uptake and DSD. Furthermore, the study relied on voluntary participation, which may have excluded individuals who were less willing or able to share their experiences, potentially limiting the representation of those who are marginalised or disengaged from care. Donmoyer (2000) notes that generalisability is not the primary aim of qualitative inquiry; instead, the focus is on depth, meaning and contextual understanding within a specific setting. Despite these limitations, the context-specific insights generated from this urban setting can inform the adaptation and strengthening of ART and DSD programmes in similar urban health systems and provide a basis for further comparative studies in other regions of Ethiopia.

#### Participant-driven recommendations

Participants proposed multi-level, integrated, person-centred solutions aligned with global best practices. They emphasised the need to ensure reliable ART drug availability by improving procurement and forecasting systems, strengthening supply-chain management and addressing infrastructure gaps that contribute to stockouts (WHO 2025). They also recommend reducing waiting times, improving appointment systems and strengthening laboratory capacity to make clinic visits more efficient and less burdensome for PLHIV. Participants further called for clear eligibility criteria for DSD models, consistent communication between providers and patients and uniform national guidelines to standardise DSD implementation (Ministry of Health, Ethiopia 2023). They recommended integrating mental health services into routine HIV care, expanding access to counselling, and supporting peer-led stigma-reduction programmes to address the psychosocial burden of living with HIV (Thalia 2024). At the provider level, they highlighted the need for ongoing training, supportive supervision and retention strategies to strengthen the quality and sustainability of HIV programmes (Ministry of Health, Ethiopia 2024).

## Conclusion

This study demonstrates that the implementation, uptake and effectiveness of DSD models for ART in Addis Ababa are shaped by a range of interconnected structural, socioeconomic and psychosocial factors, including medication stockouts, financial and logistical constraints, stigma, mental health challenges and clinic-level conditions such as waiting times, staffing shortages and communication gaps. These findings highlight the context-specific nature of HIV service delivery, where elements that facilitate DSD in one setting can act as barriers in another, and offer important insights for programme managers, policymakers, healthcare providers and researchers involved in designing and scaling person-centred interventions. However, gaps remain in understanding how specific contextual conditions, such as supply-chain robustness, stigma environments and workforce capacity, shape the success or failure of DSD models, underscoring the need for future research to adopt implementation-science frameworks and larger, context-specific studies that unpack the relative importance of different factors, identify mechanisms driving successful DSD adoption and determine which models work best for which patient groups under which conditions, thereby strengthening DSD implementation strategies and supporting the evolution of HIV programmes in Ethiopia towards more sustainable, equitable and patient-centred care.
